# Comparison of the effectiveness of ISJ and SSR markers and detection of outlier loci in conservation genetics of *Pulsatilla patens* populations

**DOI:** 10.7717/peerj.2504

**Published:** 2016-11-02

**Authors:** Katarzyna Bilska, Monika Szczecińska

**Affiliations:** Department of Botany and Nature Protection, University of Warmia and Mazury in Olsztyn, Olsztyn, Poland

**Keywords:** Microsatellites, Intron-exon splice junction (ISJ), Neutral markers, Adaptive markers, *Pulsatilla patens*, Outlier loci

## Abstract

**Background:**

Research into the protection of rare and endangered plant species involves genetic analyses to determine their genetic variation and genetic structure. Various categories of genetic markers are used for this purpose. Microsatellites, also known as simple sequence repeats (SSR), are the most popular category of markers in population genetics research. In most cases, microsatellites account for a large part of the noncoding DNA and exert a neutral effect on the genome. Neutrality is a desirable feature in evaluations of genetic differences between populations, but it does not support analyses of a population’s ability to adapt to a given environment or its evolutionary potential. Despite the numerous advantages of microsatellites, non-neutral markers may supply important information in conservation genetics research. They are used to evaluate adaptation to specific environmental conditions and a population’s adaptive potential. The aim of this study was to compare the level of genetic variation in *Pulsatilla patens* populations revealed by neutral SSR markers and putatively adaptive ISJ markers (intron-exon splice junction).

**Methods:**

The experiment was conducted on 14 Polish populations of *P. patens* and three *P. patens* populations from the nearby region of Vitebsk in Belarus. A total of 345 individuals were examined. Analyses were performed with the use of eight SSR primers specific to *P. patens* and three ISJ primers.

**Results:**

SSR markers revealed a higher level of genetic variation than ISJ markers (*H_e_* = 0.609, *H_e_* = 0.145, respectively). An analysis of molecular variance (AMOVA) revealed that, the overall genetic diversity between the analyzed populations defined by parameters *F_ST_* and Φ_*PT*_ for SSR (20%) and Φ_*PT*_ for ISJ (21%) markers was similar. Analysis conducted in the *Structure* program divided analyzed populations into two groups (SSR loci) and three groups (ISJ markers). Mantel test revealed correlations between the geographic distance and genetic diversity of Polish populations of *P. patens* for ISJ markers, but not for SSR markers.

**Conclusions:**

The results of the present study suggest that ISJ markers can complement the analyses based on SSRs. However, neutral and adaptive markers should not be alternatively applied. Neutral microsatellite markers cannot depict the full range of genetic variation in a population because they do not enable to analyze functional variation. Although ISJ markers are less polymorphic, they can contribute to the reliability of analyses based on SSRs.

## Introduction

According to recent estimates, endangered plant species account for 6% of globally identified flora (International Union for Conservation of Nature, 2014). For threatened species to be effectively protected, the existing populations have to be identified and their genetic variation has to be preserved. High genetic variation enables populations to adapt to changing habitat conditions (Hedrick, 2004; Jump & Peñuelas, 2005; Sgrò, Lowe & Hoffmann, 2011). It contributes to reproductive success and species survival (Ouborg, Vergeer & Mix, 2006). Every species is characterized by unique genetic variation which determines its ability to adapt to changing environmental conditions. Different allele combinations are responsible for adaptation to different conditions. Populations characterized by high genetic variation and abundance of diverse alleles have greater chances of survival in a changing environment (Frankham, 2003; Frankham, 2005; Kramer & Havens, 2009; Ouborg et al., 2010).

Research into the protection of rare and endangered plant species involves genetic analyses to determine their genetic variation and genetic structure. Various categories of genetic markers are used for this purpose: allozymes (Krzakowa & Michalak, 2007; López-Pujol, Zhang & Ge, 2008; Trapnell, Hamrick & Negrón-Ortiz, 2012; Dostálek, Münzbergová & Plačková, 2014), amplified fragment length polymorphism (AFLP) markers (Jian et al., 2010; Cires, Cuesta & Fernández Prieto, 2013; Wróblewska, 2013), random amplified polymorphic DNA (RAPD) markers (Wróblewska et al., 2003; Hensen, Oberprieler & Wesche, 2005; Hensen et al., 2010), restriction fragment length polymorphism (RFLP) markers (Schlögl, Souza & Nodari, 2007; Jadwiszczak et al., 2012; Jian & Zhu, 2013), inter simple sequence repeats (ISSR) markers (Buczkowska et al., 2010; Cires, Cuesta & Fernández Prieto, 2013; Gaafar, Al-Qurainy & Khan, 2014; Lopes et al., 2014) and single nucleotide polymorphism (SNP) markers (Bradley et al., 2013; Cipollini et al., 2013; Olsson & Korpelainen, 2013). These markers enable estimation of important genetic variation parameters (allelic richness, heterozygosity *H*, fixation index *F_is_*, pairwise *F_ST_*). They are used to describe past and present evolutionary processes in a given population (effective population size, bottleneck, founder effect and genetic drift). Molecular markers are also used to evaluate historical and geographic similarities between groups (Hedrick, 2001).

Microsatellites, also known as simple sequence repeats (SSR), are the most popular category of markers in population genetics research (Goldstein & Schlötterer, 1999; Somme et al., 2012). Microsatellites are codominant markers which are ubiquitous in prokaryotic and eukaryotic organisms and are characterized by high polymorphism and a high number of alleles per locus. However, they are highly species-specific and have to be isolated de novo for most species (Zane, Bargelloni & Patarnello, 2002; Abdelkrim et al., 2009).

Microsatellites are found in transcribed regions, including in protein coding genes and expressed sequence tags (EST) (Jarne & Lagoda, 1996; Morgante, Hanafey & Powell, 2002; Varshney, Graner & Sorrells, 2005). They may influence the regulation and function of some genes at both transcription and translation level (Li et al., 2002; Li et al., 2004; Varshney, Graner & Sorrells, 2005). Therefore, microsatellites may play a very important role in the genome and can even contribute to its evolution (Moxon & Wills, 1999). In most cases, microsatellites account for a large part of the noncoding DNA and exert a neutral effect on the genome. Neutrality is a desirable feature in evaluations of genetic differences between populations, but it does not support analyses of a population’s ability to adapt to a given environment or its evolutionary potential (Kirk & Freeland, 2011). Moreover, neutral markers cannot be used to analyze interactions between environmental and genetic factors that determine adaptation (Ouborg et al., 2010). Due to their high mutation rate, SSR markers are not suitable for describing evolutionary processes in the distant past. Microsatellites can be used to analyze evolutionary mechanisms such as gene flow and genetic drift (Powell, Machray & Provan, 1996; Oliveira et al., 2006). The intensity of those processes should be described to maximize the effectiveness of conservation projects, in particular those aiming to translocate individuals and restore species (Storfer, 1999; Hedrick, 2014).

An interesting alternative to the DNA markers discussed above are semi-specific Intron-exon splice junction (ISJ) markers, based on sequences commonly found in plants and indispensable for post-transcription DNA processing (Weining & Langridge, 1991). ISJ markers can be used to provide new insights in analyses based on microsatellites. These markers are dominant and the primers are partly complementary to the sequences on the exon-intron boundary (Weining & Langridge, 1991; Rafalski, 1997); therefore, amplification products can contain fragments of both coding and non-coding sequences. The amplified sequences may include fragments of functional genes, which is why ISJ markers might be regarded as selectively non-neutral markers. The sequencing of ISJ amplicons of dieocious moss *Nyholmiella obtusifolia* (Orthotrichaceae, Bryophyta), revealed that over 73% of them contain partial exonic and intronic regions (J. Sawicki & M. Ślipiko, 2014, unpublished data). The ISJ amplified bands are treated as single dominant loci and scored either present or absent.

Therefore, ISJ markers can be used to investigate adaptation processes and evolution of adaptive traits. Adaptive markers undergo selection and are used mostly in analyses of evolutionary potential, including local adaptations and speciation (Landguth & Balkenhol, 2012). Adaptive variation is responsible for the survival of a population in a changing environment and, consequently, the survival of a species (Hedrick, 2004; Kirk & Freeland, 2011). Genetic foundations of functional variation are a very important consideration in conservation genetics, in particular in studies of rare and endangered species (Gebremedhin et al., 2009; Landguth & Balkenhol, 2012).

ISJ markers are dominant, and the amplified sequences are highly conserved. One of the advantages of ISJ markers is that they do not require prior knowledge of the DNA sequence of the analyzed species (Sawicki & Szczecińska, 2007). They are not species-specific and can be used to evaluate genetic variation in different taxa. This is a considerable advantage in studies of rare and endangered species whose sequences are generally unknown. ISJ markers have been successfully used in genetic analyses of *Polygonatum* Mill. (*Asparagaceae*) (Szczecińska et al., 2006), *Aneura* Dumort. (*Aneuraceae*) (Bączkiewicz et al., 2008), *Chamaedaphne* Moench (*Ericaceae*) (Szczecińska et al., 2009), *Oryza* L. (*Poaceae*) (Hashemi-Petroudi et al., 2010) and *Sphagnum* L. (*Sphagnaceae*) (Sawicki & Szczecińska, 2011).

Despite numerous advantages of microsatellites, non-neutral markers may supply important information in conservation genetics research. They are used to evaluate adaptation to specific environmental conditions and a population’s adaptive potential, which is particularly valuable in studies of endangered species. Selected loci are likely to group populations based on adaptively relevant ecological variables, whereas neutral loci are likely to group populations phylogenetically based on geographic proximity (Nosil, Funk & Ortiz-Barrientos, 2009); therefore, loci that undergo selection provide more accurate information about genetic structure than other loci. Loci that undergo selection can also be used to investigate genetic clines along environmental gradients and “tension” hybrid zones (Guichoux et al., 2013). They are also used in evolutionary studies.

The aim of this study was to: (a) compare the values of genetic diversity parameters and genetic structure of *P. patens* populations revealed by ISJ and SSR markers; (b) detect of presence of outlier loci in the data sets; (c) examine the influence of outlier loci on the genetic structure.

In particular, we hypothesized that the ISJ markers will be less polymorphic than SSR markers. We expect that the grouping populations based on ISJ markers will be different from groupings based on SSR markers.

## Materials and Methods

### Plant material and DNA extraction

The study was conducted on *Pulsatilla patens* populations colonizing the west edge of its geographic range. Eastern pasqueflower (*Pulsatilla patens* (L.) Mill.) is a long-lived monoecious perennial herb classified as a hemicryptophyte (Kalliovirta, Ryttäri & Heikkinen, 2006). The plant blooms in early spring and produces purple-blue flowers (Wójtowicz, 2000).

*Pulsatilla patens* is an endangered plant species with a circumpolar distribution (Hultén & Fries, 1986). It appears in Asia, North America and the north-west part of Europe (Wildeman & Steeves, 1982; Dutton, Keener & Ford, 1997; Wang & Bartholomew, 2001). *Pulsatilla patens* is protected as a rapidly declining species in all European countries in which it occurs. It is listed in Appendix I of the Berne Convention (Council of Europe, 1979) and in Annexes II and IV of the Habitats Directive (Council of the European Union, 1992).

*Pulsatilla patens* grows in dry and sunny areas. In Poland, it is associated with boreal forests of the *Vaccinio-Piceetea* class (Matuszkiewicz, 2001) where it grows across wood paths, railway tracks, power lines, etc. It is often found in areas characterized by disturbed undergrowth vegetation and creates favorable conditions for germination and growth (Uotila, 1969).

**Figure 1:**
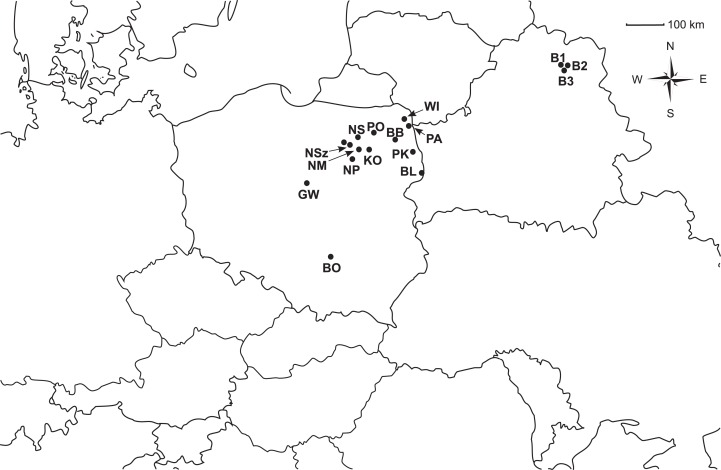
Geographic distribution of the analyzed populations of *Pulsatilla patens*.

The experiment was conducted on 14 Polish populations of *P. patens* and three *P. patens* populations from the nearby region of Vitebsk in Belarus (Table S1; Fig. 1). A total of 345 individuals were examined. The Polish populations were collected with the permission given by Polish Minister of Environment and Regional Directorates of Environmental Protection. Total genomic DNA was extracted from plant material. Leaves were grated in Mini-Beadbeater tissue disruptor and processed with the use of the Genomic Mini AX Plant SPIN kit (A&A Biotechnology) according to the manufacturer’s protocol.

### Molecular analysis

SSR analyses were performed with the use of eight primers specific to *P. patens* (Table S2) and described by Szczecińska et al. (2013). PCR reactions were performed in 20 μL of a reaction mixture containing 40 ng genomic DNA, 1x PCR buffer, 1.5 mM MgCl_2_, 200 μM dNTP (dATP, dGTP, dCTP, dTTP), 1 μL BSA, 1.0 μM of each primer and 1 U RedTaq polymerase (Sigma, St. Louis, USA). Reactions were performed under the following thermal conditions: (1) initial denaturation—4 min at a temperature of 94 °C; (2) denaturation—45 s at 94 °C; (3) annealing—50 s at 52 °C for *Pul*05, *Pul*06, *Pul*07 primers, at 55 °C for *Pul*02 primer and at 58 °C for *Pul*01, *Pul*04, *Pul*10, *Pul*11 primers; (4) elongation–90 s at 72 °C; (5) final elongation—7 min at 72 °C. Stages 2–4 were repeated 40 times.

ISJ analyses were performed using three primers (Table S2). The reaction mixture were congruent with SSR markers, except for the use of 1x PCR buffer containing MgCl_2_ and 1 U Taq polymerase (OpenExome). Thermal conditions were different in denaturation (60 s at 94 °C) and annealing (90 s at 49 °C) stages.

PCR products for ISJ and SSR markers were separated in the QIAxcel capillary electrophoresis system, using the Qiaxcel High Resolution Kit; with the 15–500 bp alignment marker (Qiagen) and pUC18/*Hae*III DNA size marker (Qiagen) for SSR markers, or the 15–3,000 bp alignment marker (Qiagen) and the 100–2,500 bp DNA size marker (Qiagen) for ISJ markers. Standard OM500 settings were used as the electrophoresis program.

The size of the obtained SSR and ISJ bands were determined by using BioCalculator softwere (Qiagen).

### Data analysis

Allele frequency in each amplified ISJ locus was identified in view of band presence or absence. It was assumed that every observed band resulted from the amplification of a single locus, therefore, the number of observed bands corresponded to the number of investigated loci. Only allele “1” (band present) or allele “0” (band absent) were observed in every locus. The presence of null alleles was checked using the MICRO-CHECKER 2.2.3 program (van Oosterhout et al., 2004). For each population/locus we calculated frequency of null alleles using Brookfield’s estimator 2 (Brookfield, 1996).

The genetic diversity parameters, including the number of alleles per locus (*A*), the number of effective alleles (*A_e_*), observed heterozygosity (*H_o_*), expected heterozygosity (*H_e_*), fixation index *F_is_*, the number of loci (*N*), the number of polymorphic loci (*n*) and the percentage of polymorphic loci (*P*), were generated by use of *GenAlEx v. 6.501* software (Peakall & Smouse, 2006; Peakall & Smouse, 2012). Linear regression implemented in *Statistica v. 12* (StatSoft Inc., Tulsa, OK, USA) was performed to test correlation between measured parameters and the populations size. Tests of deviation from Hardy–Weinberg proportions at each SSR locus Hardy–Weinberg equilibrium (HWE) and of linkage disequilibrium between pairs of loci were tested in *FSTAT v.2.9.3* (Goudet, 1995).

BayeScan v. 2.1 software (Foll & Gaggiotti, 2008) and the implemented R function were used to identify candidate loci under natural selection (outlier loci) in genetic data based on differences in allele frequencies between populations. The default parameters given in the program were used. Further in the paper, we use the term ‘neutral loci’ (non-outliers) with reference to loci indicated by BayeScan as not under natural selection. Another reason for such nomenclature was to distinguish these loci from outlier ones. Thus, we can assume that both neutral SSR and neutral ISJ loci are probably under genetic drift.

Genetic distance between the studied populations was described by calculating pairwise *F_ST_* (Hartl & Clark, 1997) and *R_ST_* values (Slatkin, 1995) for SSR markers and Φ_*PT*_ values for ISJ markers. An analysis of molecular variance (AMOVA) was conducted to assess the partitioning of the genetic variance within and among populations. AMOVA was performed using *F_ST_* and Φ_*PT*_ values for SSR markers and Φ_*PT*_ values for ISJ markers. The possible correlations between genetic and geographic distance (isolation by distance) (Slatkin, 1993; Excoffier, Laval & Schneider, 2005) were estimated for Polish populations by correlating *F_ST_*/(1 − *F_ST_*) with geographic distance (km) in a Mantel test (Mantel, 1967). Coordinates for Belorussian populations were unavailable, thus those populations were excluded from Mantel test analysis. AMOVA and the Mantel test were run for 9,999 permutations to assess significance. Analyses were performed in *GenAlEx v.6.501* (Peakall & Smouse, 2006; Peakall & Smouse, 2012).

Principal coordinate analysis (PCoA) based on the pairwise *F_ST_* distance matrix (for SSR) and Φ_*PT*_ distance matrix (for ISJ) was carried out in GenAIEx *6.501* software (Peakall & Smouse, 2006; Peakall & Smouse, 2012). The analysis was performed for all detected SSR and ISJ loci, and separate for neutral (non-outlier) and outlier loci.

Population structure was analyzed by Bayesian clustering provided by the *Structure v. 2.3.4* software (Pritchard, Stephens & Donnelly, 2000). Simulations were performed under an admixture model on the assumption of correlated allele frequencies. In the preliminary analysis, the possible number of tested clusters (K) ranged from 1 to 18 (the putative number of populations plus one). The analysis was performed for 10 iterations, 10,000 burn-in period and 100,000 MCMC repetitions after burning. The most likely number of populations (K) was identified using the Delta K method (Evanno, Regnaut & Goudet, 2005) implemented in *Structure Harvester* (Earl & vonHoldt, 2012). Subsequent analyses were performed for a selected number of clusters with 100,000 burn-in period and 1000,000 MCMC repetitions after burning. *Structure* results were summarized using the *CLUMPAK* server (Kopelman et al., 2015) to obtain the probability of each individual to belong to each cluster. The number of groups were chosen after the *Structure* output files were analyzed in R software (R Core Team, 2014). Similarity among results of different runs for the same K was calculated according to Nordborg et al. (2005) using *Structure-sum 2009* (Ehrich et al., 2007). The percentage membership of each individual in every cluster was determined by the value of Q, and each individual was assigned to a specific cluster based on an arbitrary threshold of Q > 0.75. The genetic structure analysis was performed for all detected loci SSR and ISJ, and separately for neutral loci (non-outlier) and outlier ones.

## Results

### Detection of outlier loci

**Figure 2:**
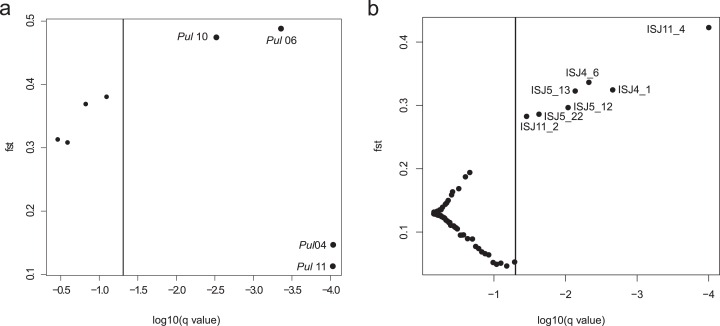
*BayeScan* plots. (A) SSR markers; (B) ISJ markers.

An analysis of ISJ markers in the *BayeScan* program revealed seven outlier loci in the group of 75 amplified loci (Fig. 2B). All detected outlier loci had positive alpha value and high value of *F_ST_* (*F_ST_* = 0.284–0.424; Table S3).

**Figure 3:**
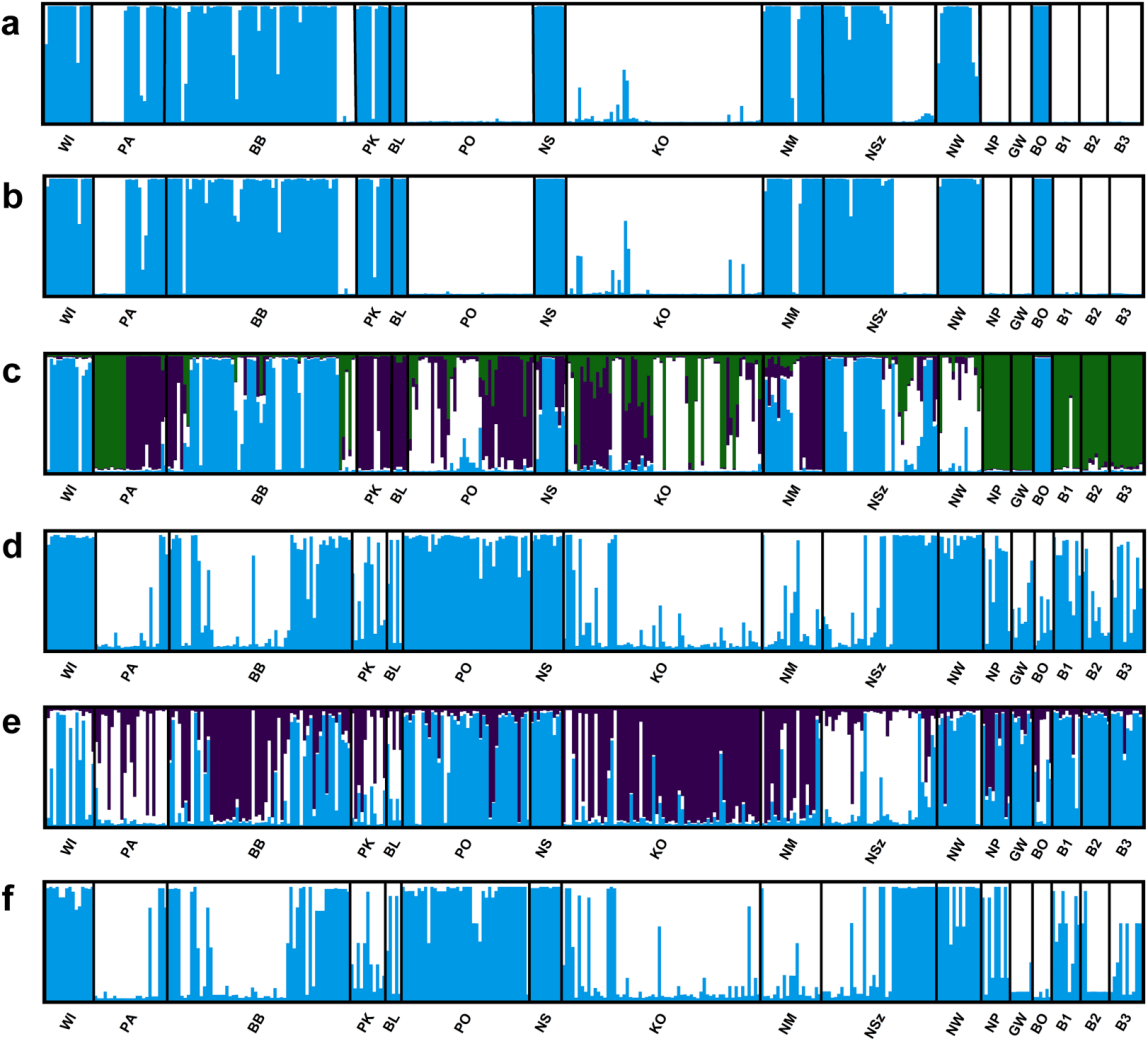
Genetic structure of *P. patens* populations. (A) Based on all studied SSR loci at K = 2; (B) based on neutral SSR loci at K = 2; (C) based on outlier SSR loci at K = 4; (D) based on all studied ISJ loci at K = 2; (E) based on neutral ISJ loci at K = 3; (F) based on outlier ISJ loci at K = 2.

**Figure 4:**
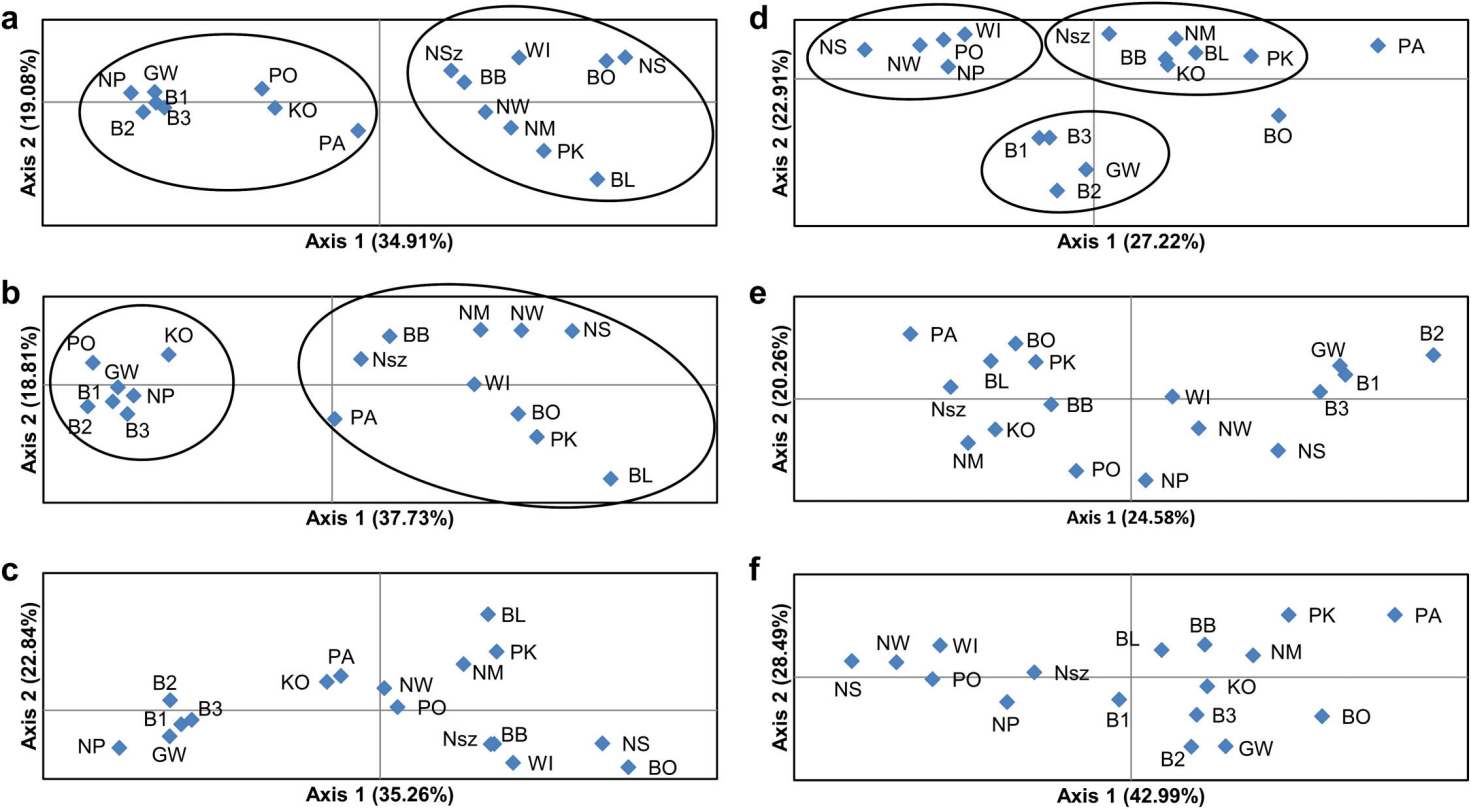
Principal coordinate analysis (PCoA) plots of *P. patens* populations. (A) Based on all studied SSR loci; (B) based on neutral SSR loci; (C) based on outlier SSR loci; (D) based on all studied ISJ loci; (E) based on neutral ISJ loci; (F) based on outlier ISJ loci.

Four of the eight evaluated SSR loci were outlier loci (*F_ST_* = 0.113–0.488; Table S3). Two of them had negative alpha value. The elimination of those four outlier loci from analysis in the *Structure* program and PCoA analysis had no significant influence on the genetic structure of the evaluated populations of Eastern pasqueflower (Figs. 3 and 4).

### Genetic diversity

A total of 92 alleles were identified across eight SSR loci in 345 individuals (Table S2). The presence of null alleles were observed in most analyzed loci. The highest number of alleles (18) was amplified by primers *Pul*11, whereas *Pul*2, *Pul*6 and *Pul*10 produced the lowest value of alleles (8). Three ISJ markers revealed altogether 75 loci (Table S2). All applied primers amplified similar number of loci: ISJ4-27 loci, ISJ5-23 and ISJ11-25 loci.

SSR markers revealed a much higher level of genetic variation than ISJ markers. The genetic diversity indices for eight microsatellite loci and ISJ markers calculated for each population are given in Table 1.

**Table 1:** Genetic variation of *P. patens*.

	SSR (all loci)						SSR (outlier loci)						ISJ (all loci)					ISJ (outlier loci)				
Population	*A*	*A_e_*	*P* (%)	*H_o_*	*H_e_*	*F_is_*	*A*	*A_e_*	*P* (%)	*H_o_*	*H_e_*	*F_is_*	*N*	*n*	*P* (%)	*A_e_*	*H_e_*	*N*	*n*	*P* (%)	*A_e_*	*H_e_*
**WI**	5.1	3.48	100	0.150	0.657	0.821	5.5	4.1	100	0.300	0.699	0.643	36	36	100	1.288	0.165	5	5	100	1.364	0.216
**PA**	5.8	4.41	100	0.141	0.737	0.832	6.7	5.0	100	0.283	0.740	0.655	48	47	97.91	1.305	0.178	6	6	100	1.699	0.360
**BB**	8.7	4.62	100	0.180	0.735	0.794	10.2	5.1	100	0.360	0.726	0.588	55	55	100	1.277	0.171	7	7	100	1.699	0.376
**PK**	3.6	2.37	100	0.138	0.535	0.801	3.7	2.5	100	0.275	0.586	0.602	36	35	97.22	1.234	0.141	4	4	100	1.443	0.248
**BŁ**	1.7	1.55	62.5	0.175	0.288	0.457	2.0	1.6	75	0.350	0.345	0.095	31	23	74.19	1.158	0.098	5	4	80	1.358	0.210
**PO**	5.3	3.33	100	0.163	0.666	0.795	5.7	3.5	100	0.325	0.669	0.590	54	54	100	1.292	0.179	5	5	100	1.377	0.215
**NS**	3.2	2.18	100	0.150	0.455	0.778	3.5	2.4	100	0.300	0.515	0.556	39	32	82.05	1.165	0.105	2	1	50	1.124	0.066
**KO**	7.8	4.89	100	0.175	0.746	0.803	9.5	6.2	100	0.343	0.784	0.617	51	50	98.03	1.292	0.175	7	7	100	1.688	0.391
**NM**	4.5	3.02	100	0.151	0.644	0.801	5.0	3.4	100	0.263	0.673	0.666	45	44	97.77	1.290	0.174	7	7	100	1.704	0.398
**NSz**	7.0	4.93	100	0.201	0.763	0.764	7.2	5.1	100	0.403	0.745	0.528	49	48	97.95	1.281	0.176	6	5	83.33	1.390	0.244
**NW**	4.3	3.14	100	0.214	0.611	0.740	5.2	3.7	100	0.429	0.631	0.479	34	34	100	1.238	0.142	3	3	100	1.295	0.164
**NP**	4.5	3.20	100	0.181	0.589	0.773	5.2	3.5	100	0.361	0.579	0.546	34	28	82.35	1.228	0.132	5	5	100	1.497	0.274
**GW**	4.1	3.41	100	0.161	0.630	0.813	5.2	4.3	100	0.321	0.668	0.625	37	28	75.67	1.190	0.114	5	2	40	1.268	0.138
**BO**	2.7	2.20	87.5	0.167	0.458	0.613	2.5	1.7	100	0.292	0.420	0.397	45	39	86.66	1.269	0.163	7	5	71.43	1.561	0.299
**B1**	4.1	3.12	100	0.139	0.644	0.822	4.2	3.3	100	0.278	0.647	0.643	30	25	83.33	1.185	0.110	5	4	80	1.408	0.228
**B2**	4.1	2.78	100	0.167	0.550	0.763	4.7	3.1	100	0.333	0.520	0.526	33	24	72.72	1.215	0.123	5	3	60	1.370	0.198
**B3**	4.3	3.47	100	0.213	0.646	0.750	5.5	4.3	100	0.425	0.688	0.449	33	25	75.75	1.208	0.120	5	4	80	1.528	0.274
**At species level**	**4.7**	**3.30**	97.06	**0.169**	**0.609**	**0.768**	**5.4**	**3.7**	98.53	**0.332**	**0.626**	**0.552**	**75**	**75**	**100**	**1.242**	**0.145**	**7**	**7**	**100**	**1.454**	**0.253**
SD	0.243	0.2	2.28	0.029	0.017	0.040	4.2	2.8	1.47	0.051	0.025	0.069				0.009	0.005				0.038	0.020

**Notes:**

*A*, number of alleles per locus; *A_e_*, effective number of alleles; *H_o_*, observed heterozygosity; *H_e_*, expected heterozygosity; *F*_*is*_, fixation index; *N*, number of loci; *n*, number of polymorphic loci; *P*, percentage of polymorphic loci; SD, standard deviation.

The average expected heterozygosity reached *H_e_* = 0.609 when estimated with SSR markers (all loci) and *H_e_* = 0.145 when estimated with ISJ markers (all loci) (Table 1). A detailed analysis of the genetic diversity parameters at the population level show some differences for both categories of used markers. For SSR markers (analysis of all loci) the highest average number of alleles and mean expected heterozygosity were found in NSz population (*A_e_* = 4.93, *H_e_* = 0.763) (Table 1). For ISJ, *A_e_* was highest in population PA (Augustów; *A_e_* = 1.305) and *H_e_* was highest in populations PA (Augustów; *H_e_* = 0.178) and PO (Orzysz; *H_e_* = 0.179).

Analysis of genetic diversity parameters only for outlier SSR loci generally didn’t show differences in comparison to the results for all loci. In contrast, very large differences were observed for outlier ISJ loci. The value of *H_e_* calculated for outlier ISJ loci was twice as high compared to the results for all loci (Table 1).

**Figure 5:**
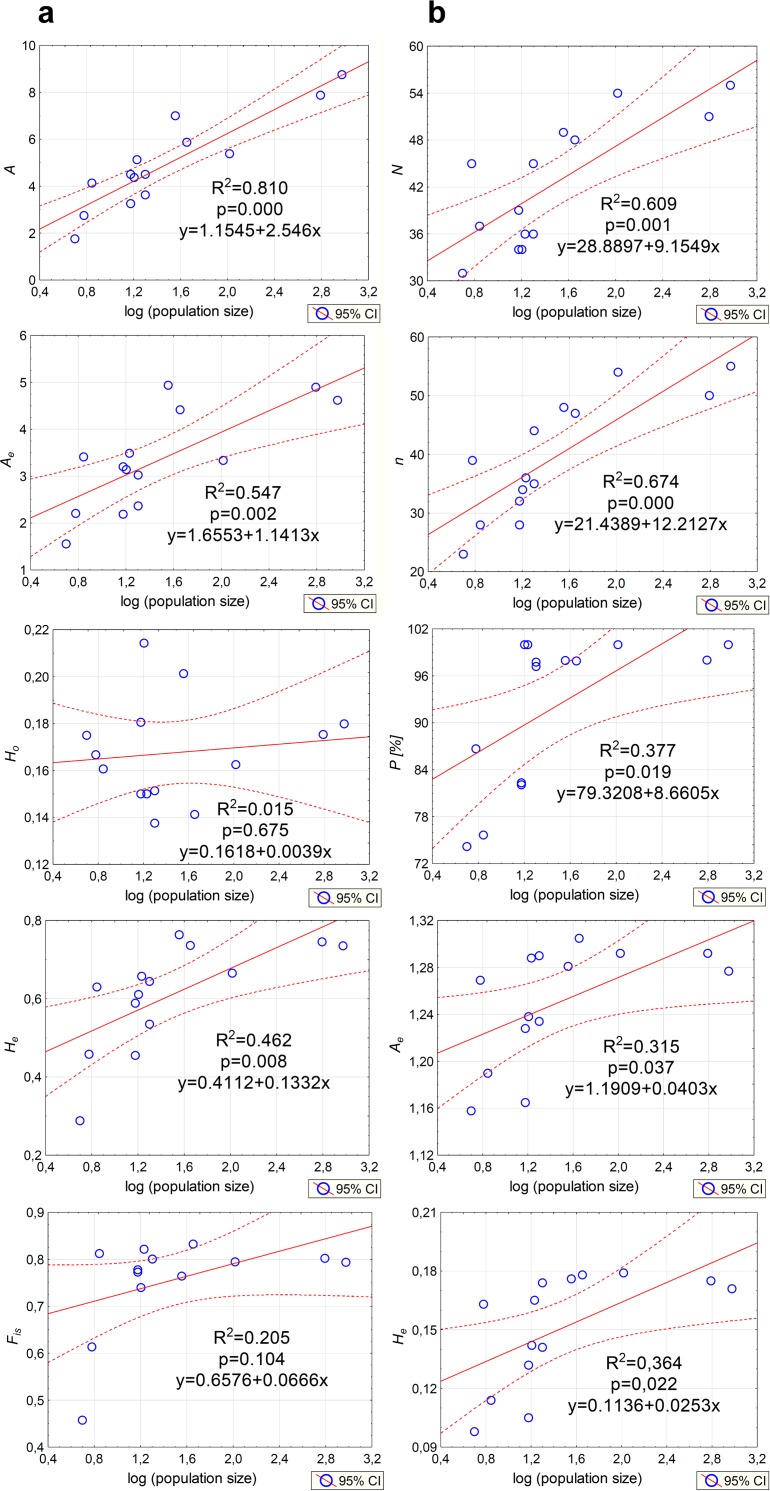
Correlations between the size of the analyzed populations of *P. patens* and genetic variation parameters. (A) Based on SSR markers; (B) based on ISJ markers.

Genetic variation parameters *A*, *A_e_*, *H_e_* calculated for microsatellite loci were positively correlated with population size (Fig. 5A). For ISJ markers, a positive correlation was observed for all evaluated parameters (*n*, *P*, *A_e_*, *H_e_*; Fig. 5B).

### Genetic structure

AMOVA revealed that, the overall genetic diversity between the analyzed populations defined by parameters *F_ST_* and Φ_*PT*_ for SSR (20%) and Φ_*PT*_ for ISJ (21%) markers was similar (Table S4). This implies that the main component of variation was part of intrapopulation variation.

An analysis of genetic diversity parameters (*F_ST_*, *R_ST_*-SSR and Φ_*PT*_-ISJ) revealed differences in their extreme values for both categories of applied markers (Tables S5 and S6). In SSR analysis, the greatest genetic similarity was observed between pairs of populations: BB (Biebrza)–NSz (Szczytno) (*F_ST_* = 0.069; Table S5), BB (Biebrza)–PO (Orzysz) (*R_ST_* = 0.024) and BB (Biebrza)–NSz (Szczytno) (*R_ST_* = 0.024). The highest level of genetic differentiation were noted between: populations NP (Parciaki)–BL (Białowieża) *F_ST_* = 0.475), and populations GW (Gostynin-Włocławek)–BL (Białowieża) (*R_ST_* = 0.901), and populations GW (Gostynin-Włocławek)–NS (Strzałowo) (*R_ST_* = 0.901).

For ISJ markers the highest level of similarity (Φ_*PT*_) was noted between populations KO (Kolimagi)–NM (Myszyniec) (Φ_*PT*_ = 0.044; Table S6), whereas the highest genetic differences were found between populations PA (Augustów)–NS (Strzałowo) (Φ_*PT*_ = 0.405).

The above differences were validated by the results of the Mantel test which did not reveal correlations between the geographic distance and genetic diversity of Polish populations of *P. patens* for SSR markers (for *F_ST_*: *R*^2^ = 0.126 and *p* = 0.239, for *R_ST_*: *R*^2^ = 0.002 and *p* = 0.440). The presence of such correlations was noted for ISJ markers based on parameter Φ_*PT*_ (*R*^2^ = 0.144, *p* = 0.003).

Both categories of markers revealed together 14 specific alleles in analyzed populations in each case. SSR markers amplified eight specific alleles and ISJ markers–six specific allels (Table S2). This alleles were noted in populations: BB (Biebrza), KO (Kolimagi), NP (Parciaki), BO (Bocheniec) and B2 (Vitebsk, Belarus, pop. 2), whereas for ISJ those alleles were found in populations: PO (Orzysz), KO (Kolimagi) and NSz (Szczytno). Both categories of markers amplified specific alleles only in population KO (Kolimagi).

Depending on the data used (all analyzed SSR and ISJ loci, outlier SSR and ISJ loci, only neutral SSR and ISJ loci) the analysis conducted in the *Structure* program revealed a different number of clusters for studied populations (Table S8; Figs. 3 and S7). The presence of two genetic groups (K = 2, Evanno methods) was identified in *P. patens* individuals for all detected SSR loci, neutral SSR loci, for all detected ISJ loci and outlier ISJ loci. However, depending on the data used, particular populations differ in membership of each of these two groups. Other numbers of genetic cluster was identified for outlier SSR loci (K = 4; Fig. 3C) and neutral ISJ loci (Fig. 3E).

PCoA based on Nei’s unbiased genetic distance (Fig. 4) generally didn’t confirm the separation of *Pulsatilla patens* populations as inferred by the Bayesian approach. Two main groups of populations were found only for analysis conducted for all SSR loci and neutral SSR loci (Figs. 4A and 4B). Whereas differences were also observed in every population’s membership in each group. In the case of other PCoA analysis the number of selected groups were different from the Bayesian approach. The distribution of the analyzed populations in PCoA diagrams does not correspond to their geographic distribution.

## Discussion

The applied categories of markers, SSR and ISJ, revealed differences in the degree and distribution of genetic variation in the analyzed populations of *Pulsatilla patens*. In view of the specific traits of the applied markers, genetic variation was generally average (SSR *H_e_* = 0.609, ISJ *H_e_* = 0.145; Table 1).

Microsatellites revealed higher heterozygosity than ISJ markers (Table 1), but these differences can be attributed to the unique properties of SSR markers: codominance, high mutation rate and high polymorphism. Lower polymorphism of dominant markers in comparison with codominant markers was confirmed before in other studies (Garcia et al., 2004; van Treuren & van Hintum, 2009; Li et al., 2011; Hamza et al., 2013). Lower variation of ISJ markers can also result from their conserved sequences. Similar results were reported by D’Esposito et al. (2012) in seagrass species of *Posidonia oceanica* (L.) Delile. The cited authors relied on SSR and EST-SSR markers and found that SSR revealed a higher number of alleles per locus (*A* = 2.077–4.044) and higher expected heterozygosity (*H_e_* = 0.293–0.485) than EST-SSR (*A* = 1.255–1.431, *H_e_* = 0.069–0.121). Coscia et al. (2012) also reported higher heterozygosity values for SSRs (*H_e_* = 0.675–0.718, *H_o_* = 0.644–0.736) than for adaptive EST-SSR markers (*H_e_* = 0.634–0.663, *H_o_* = 0.561–0.629). Singh et al. (2013), however, observed very similar levels of genetic variation based on putatively neutral SSRs and SNPs developed from conserved single-copy rice genes (2.2/2.0 alleles per primer, 0.30/0.28 gene diversity, 0.12/0.19 heterozygosity). The cited authors did not test the neutrality of the applied markers.

It is worth to mention that distinctive differences in genetic variation of the studied populations were noted between the applied markers. The populations, where the analyzed parameters reached the highest and lowest values did not correspond for both categories of markers. This could suggest that the ISJ markers used in this study were not selectively neutral. The observed differences could be attributed to the fact that neutral markers are affected by genetic drift, whereas adaptive markers by selection. In the studied populations, the balance between selection and genetic drift is probably disrupted, as demonstrated by the differences in the values of genetic variation parameters.

At present, the exclusive use of neutral markers in evaluations of genetic variation is often questioned (Holderegger, Kamm & Gugerli, 2006; Marsden et al., 2012), in particular in populations of endangered species. Adaptive variation is more important for the survival of a population under changing environmental conditions (Hedrick, 2004; Kirk & Freeland, 2011). In the above studies, a higher level of genetic variation was noted based on SSR markers, but it does not necessarily reflect the adaptive potential of the analyzed populations. Populations characterized by a high level of genetic variation estimated based on ISJ markers probably have higher adaptive potential. Despite the above, genes responsible for present and past adaptations may not play a key role in future adaptive processes (Allendorf, Hohenlohe & Luikart, 2010; Funk et al., 2012). For this reason, variations should be identified in both neutral genes and genes that undergo selection.

The applied categories of molecular markers did not reveal specific genetic structure in the analyzed populations of *P. patens*. The distribution of genetic variation was generally similar for both categories of used markers. The absence of distinctive differences between the analyzed populations can be attributed to the fact that until recently, Eastern pasqueflower had continuous geographic range in Eastern and Central Europe (Hultén & Fries, 1986; Akeroyd, 1993). Nowadays the populations of the studied species are gradually disappearing and becoming partially isolated (Ciosek, 1999; Wójtowicz, 2000; Wójtowicz, 2001; Chmura, 2003; Wójtowicz, 2004; Juśkiewicz-Swaczyna, 2010). However, this process has not been taking place long enough for evolutionary mechanisms to induce significant differences between populations. The absence of a clear genetic structure was documented by AMOVA which revealed much higher percentage of variation within a population than between populations for both categories of markers (Table S4). Our results suggest that both markers are equally useful for evaluating the hierarchical distribution of intrapopulation and interpopulation variation. Singh et al. (2013) reported a very similar distribution of variation based on putatively neutral SSR markers (1% among regions, 4% among populations, 70% among individuals, 25% within individual) and SNP markers developed from conserved single-copy rice genes (0% among regions, 1% among populations, 67% among individuals, 32% within individual). However, Woodhead et al. (2005) analyzed neutral SSR and AFLP markers and adaptive EST-SSR markers and reported a similar distribution of variation for both types of SSR markers (34.18 and 34.45% among countries, 7.43 and 5.22% among populations, 58.39 and 60.33% within populations for genomic SSR and EST-SSR, respectively) and different distribution for AFLP markers (57.6% among countries, 8.56% among populations, 34.32% within populations).

Differences between the evaluated marker categories were noted in the level of genetic diversity between the analyzed populations. For both categories of markers extreme values of genetic diversity parameters (*F_ST_*, *R_ST_* and Φ_*PT*_) were noted in different population pairs (Tables S5 and S6). As mentioned earlier, selection influences mostly adaptive markers, whereas genetic drift affects neutral markers. Evolutionary mechanisms in different populations contribute to changes in different parts of the genome. The rate of evolution is also different in various genome fragments. Landguth & Balkenhol (2012) demonstrated that genetic data which undergo selection reveal differences between populations more quickly than neutral data.

Despite the fact that genetic diversity parameters revealed highly extreme values between the studied populations, the average values of those parameters were moderate (*F_ST_* = 0.196, *R_ST_* = 0.108, Φ_*PT*_ = 0.209). A higher level of genetic diversity, estimated based on putatively non-neutral ISJ markers (Φ_*PT*_ = 0.209), is consistent with the results obtained by Landguth & Balkenhol (2012). Coscia et al. (2012), however, analyzed 15 SSR loci and 23 EST-SSR loci and found a higher level of genetic diversity for neutral markers (*F_ST_* = 0.068 for SSR, *F_ST_* = 0.016 for EST-SSR). Nevertheless, when two SSRs suspected of undergoing directional selection were eliminated, the value of *F_ST_* (SSR *F_ST_* = 0.011) dropped below that noted for EST-SSR loci.

The number of identified populations of a given species often does not correspond to the number of actual genetic groups. An analysis of PCoA diagrams revealed that markers split the studied populations into two groups (microsatellite loci) or three groups (ISJ loci) which, however, differed in the populations that created them (Figs. 4A and 4D). A simulation for the SSR markers in the *Structure* program also supported the identification of two genetic groups of individuals that differed in allele frequency (Figs. 3A and 3D). The proportion of the identified genetic groups in each population differed for the two markers. High genetic homogeneity for both markers was found only in populations WI (Wigry), PO (Orzysz), NS (Strzałowo), KO (Kolimagi), NM (Myszyniec), NW (Wielbark) and BO (Bocheniec) (Table S8; Fig. 3). The presence of individuals from both identified genetic groups was noted in the remaining populations, but it differed for both markers. It should be noted that for both categories of markers, the distribution of the analyzed populations in PCoA diagrams as well as the percentage of genetic groups identified in each population in the *Structure* program were uncorrelated with their geographic distribution. PCoA diagrams show differences between all SSR and all ISJ loci (Figs. 4A and 4D) and outlier ones (Figs. 4C and 4F). These differences in PCA diagrams for neutral SSR markers and putatively non-neutral markers were reported by Loywyck et al. (2008) (SSR markers in QTL regions) and Singh et al. (2013) (SNP markers developed from conserved single-copy genes), too. Furthermore, in a *Structure* analysis performed by Singh et al. (2013), the values of K were determined by the applied markers (K = 5 for SSRs, K = 15 for SNPs). Bublyk et al. (2013), however, analyzed three types of markers amplifying non-coding sequences (RAPD, ISSR, inter-retrotransposon amplified polymorphism (IRAP)) and two types of markers based on conserved gene sequences: disease resistance (resistance gene analog polymorphism (RGAP)) and abiotic stress response (long primer polymerase chain reaction (LP-PCR)), and found different grouping patterns of *Iris pumila* individuals in PCoA diagrams for each marker. An analysis of *I. pumila* populations in the *Structure* program also produced ambiguous results (Bublyk et al., 2013). A single genetic group was identified by RAPD, ISSR and LP-PCR markers, and two genetic groups were distinguished by IRAP and RGAP markers. Therefore, the results noted for markers complementary to coding sequences as well as for markers complementary to non-coding sequences were ambiguous. The differences in the grouping patterns of *P. patens* populations for SSR and ISJ markers in PCoA diagrams and the variations in the proportions of the two genetic groups identified in the studied populations in the *Structure* program could be attributed to significant differences in the values of genetic diversity parameters between populations (*F_ST_*, *R_ST_*, Φ_*PT*_). It could also point to differences in the evolution of the analyzed genome fragments. According to Funk et al. (2012), neutral and adaptive data can group populations differently depending on genetic drift, gene flow and selection. However in their opinion, the *Structure* program is not suitable for grouping populations based on adaptive loci. According to the cited authors, the algorithm implemented in the program assumes Hardy–Weinberg proportions and linkage equilibrium which are not appropriate for loci under selection (Funk et al., 2012).

The results of this study do not unambiguously support the conclusion that Polish populations of *P. patens* are undergoing isolation by distance. The Mantel test based on SSR markers did not reveal correlations between the geographic distance and genetic diversity of Polish populations of *P. patens*. A statistically significant correlation was noted for ISJ markers. Despite the above, the estimated value of the test was low (*R*^2^ = 0.144), which indicates that geographic distance was weakly correlated with genetic diversity. The Mantel test produced different results for SSRs and putatively adaptive ISJ markers, which confirms that the diversification process has only just begun in *P. patens* populations and is probably associated with the ongoing isolation of localities.

As mentioned before, genetic data that undergo selection reveal population diversity more quickly than neutral data (Landguth & Balkenhol, 2012). Despite the above, both microsatellites and ISJ markers revealed only 14 specific alleles in *P. patens* populations (Table S2). However, for both markers specific alleles were observed in different populations. Specific alleles were revealed by both SSR and ISJ loci only in population KO (Kolimagi). It should also be noted that outlier loci were not identified among the revealed specific alleles for ISJ markers. For SSR markers, one specific allele belonged to locus *Pul04* which was classified as an outlier locus. Jatoi et al. (2010) studied *Curcuma amada* farm and genbank accessions with the use of neutral (rice SSR based RAPDs) and functional genomic (P450 based analog) markers and observed that neutral markers amplified more specific alleles (78) than functional markers (63).

Microsatellites have an advantage over ISJ markers in that they support evaluations of inbreeding. Inbreeding decreases the level of genetic variation (Frankham, 2003). Inbreeding does not modify the frequency of alleles in a population, but it changes the frequency of genotypes, increases the homozygosity of all loci and decreases genetic variation (Keller & Waller, 2002; Edmands, 2007). Inbreeding and loss of genetic variation diminish population viability and contribute to the risk of extinction (Frankham, 2003; Frankham, 2005). Inbreeding applies to both neutral and functional variation. Unlike the codominant SSR markers, however, the dominant ISJ markers do not enable to analyze the level of inbreeding.

Identification of outlier loci is one of a key steps in understanding the evolutionary process, because those loci are responsible for genetic variants that affect fitness in different environment (Feng, Jiang & Fan, 2015). Outlier loci can better explain the adaptive genetic variation that is not accounted for by neutral loci (Luikart et al., 2003). Although it is expected that footprints of selection should be more frequent in ISJ than in SSRs.

Detections of outlier loci in the *BayeScan* program confirmed that both of the analyzed markers were subject to selection. Four of the eight evaluated SSR loci were outlier loci (Table S3). However the elimination of those four outlier loci from analysis had no significant influence on the genetic structure of the evaluated populations of Eastern pasqueflower (Figs. 3 and 4). The group of 75 amplified loci contained seven outlier loci, which accounted for 9.33% of the examined ISJ loci (Fig. 2; Table S3). A similar percentage of outlier loci was reported by other authors (Nosil, Egan & Funk, 2008; Manel, Conord & Després, 2009; Nosil, Funk & Ortiz-Barrientos, 2009; Freeland et al., 2010; Xia et al., 2014).

Our study confirms that the genetic structure detected based only on outlier loci and on neutral loci may be completely different. It suggests that outlier loci played a key role in the development of the genetic structure of the examined *P. patens* populations and that the analyzed ISJ markers were subject to selection. Most of the detected outlier loci had positive alpha value (Table S3), which is indicative of diversifying selection. A similar result was observed by Soto-Cerda & Cloutier (2013) for *Linum usitatissimum* L. and Feng, Jiang & Fan (2015) for *Ceracris kiangsu*.

The results of the present study suggest that ISJ markers can complement the analyses based on SSRs. This observation is consistent with the increasingly popular trend where both neutral variation and adaptive variation are analyzed (Woodhead et al., 2005; Kirk & Freeland, 2011; Coscia et al., 2012; Bublyk et al., 2013). A reliable estimation of genetic variation plays a key role in conservation genetics because it supports evaluations of the present and future populations’ conditions (Kirk & Freeland, 2011). However, neutral and adaptive markers should not be applied alternatively. Microsatellites, which are usually selectively neutral markers, can be used to investigate gene flow and genetic drift, whereas putatively non-neutral ISJ markers enable to analyze selection, adaptation and evolutionary processes. Microsatellites amplify random fragments of the genome, mostly non-coding fragments that are not responsible for inheritable traits. By contrast, ISJ markers can reveal variations in coding regions that may determine the adaptive traits of an organism. A combined analysis of functional and neutral variation produces more reliable conclusions and supports reliable decision-making in conservation genetics. Neutral microsatellite markers cannot depict the full range of genetic variation in a population because they do not enable to analyze functional variation. Although ISJ markers are less polymorphic, they can contribute to the reliability of analyses based on SSRs.

## Supplemental Information

10.7717/peerj.2504/supp-1 Supplemental Information 1 The analyzed populations of *P. patens*.

10.7717/peerj.2504/supp-2 Supplemental Information 2 Description of primers used in the study.

10.7717/peerj.2504/supp-3 Supplemental Information 3 Detection of outlier loci using *BayeScan*.

10.7717/peerj.2504/supp-4 Supplemental Information 4 Analysis of molecular variance (AMOVA) for *P. patens* populations.

10.7717/peerj.2504/supp-5 Supplemental Information 5 Genetic diversity *F_ST_* (below diagonal) and *R_ST_* (above diagonal) between the studied populations of *P. patens* based on all SSR loci.

10.7717/peerj.2504/supp-6 Supplemental Information 6 Genetic diversity Φ_*PT*_ between the studied populations of *P. patens* based on all ISJ loci.

10.7717/peerj.2504/supp-7 Supplemental Information 7 Results of *Structure* analysis–ΔK values as function of K and mean logarithmic likelihood of K values. (A) based on all studied SSR loci; (B) based on neutral SSR loci; (C) based on outlier SSR loci; (D) based on all studied ISJ loci; (E) based on neutral ISJ loci; (F) based on outlier ISJ loci.

10.7717/peerj.2504/supp-8 Supplemental Information 8 Membership of particular populations to a specific cluster based on an arbitrary threshold of Q > 0.75. Populations with Q < 0.75 for each clusters are not included.

10.7717/peerj.2504/supp-9 Supplemental Information 9 SSR and ISJ datasets for *P. patens* populations.

## Additional Information and Declarations

### Field Study Permissions

The following information was supplied relating to field study approvals (i.e., approving body and any reference numbers): The Polish populations were collected with the permission given by Polish Minister of Environment and Regional Directorates of Environmental Protection.

### Data Deposition

The following information was supplied regarding data availability: The raw data has been supplied as Supplementary Dataset Files.
