# Supplementary material for: Comparison of the effectiveness of ISJ and SSR markers and detection of outlier loci in conservation genetics of Pulsatilla patens populations
Source: PeerJ. 2016 Nov 2;4:e2504. doi: 10.7717/peerj.2504 (PMC5101595; doi:10.7717/peerj.2504)
Supplement: Supplemental Information 1 [file peerj-04-2504-s001.pdf]

Supplemental table S1. The analyzed populations of *P. patens*

	**Locality**	**Coordinates**	**Population size**	**Analyzed individuals**
WI	Wigry National Park	N: 54° 2' 10.75"<br>E: 23° 12' 22.07"	17	15
PA	Augustów Primeval Forest	N: 53° 53' 4.25”<br>E: 23° 21' 54.55"	45	23
BB	Biebrza National Park	N: 53° 34' 50.06"<br>E: 22° 51' 2.00"	944	60
PK	Knyszyn Primeval Forest Landscape Park	N: 53°15’ 12.18”<br>E: 23°29’48.07”	20	11
BL	Białowieża National Park	N: 52°47’53.75”<br>E: 23°56’12.04”	5	5
PO	Orzysz Military Area	N: 53° 44' 1.38"<br>E: 21° 59' 45.14"	104	40
NS	Strzałowo Forest Division	N: 53°38’47.43”<br>E: 21°27’40.4”	15	10
KO	Kolimagi	N: 53° 21' 39.73"<br>E: 21° 50' 51.33"	619	62
NM	Myszyniec Forest Division	N: 53°20’2.68”<br>E: 21°36’6.57”	20	19
NSz	Szczytno Forest Division	N: 53° 27' 2.15”<br>E: 21° 1' 29.82”	36	36
NW	Wielbark Forest Division	N: 53° 29' 19.50"<br>E: 20° 53' 22.67"	16	14
NP	Parciaki Forest Division	N: 53° 7' 11.65"<br>E: 21° 11' 26.81"	15	9
GW	Gostynin-Włocławek Landscape Park	N: 52°32’59,2’’<br>E: 19°24’04,4’’	7	7
BO	Bocheniec	N: 50°48’10.69”<br>E: 20°18’58.76”	6	6
B1	Vitebsk, Belarus, pop. 1	n/a	n/a	9
B2	Vitebsk, Belarus, pop. 2	n/a	n/a	9
B3	Vitebsk, Belarus, pop. 3	n/a	n/a	10
	**Total:**			**345**

n/a- data not available
