# Supplementary material for: Comparison of the effectiveness of ISJ and SSR markers and detection of outlier loci in conservation genetics of Pulsatilla patens populations
Source: PeerJ. 2016 Nov 2;4:e2504. doi: 10.7717/peerj.2504 (PMC5101595; doi:10.7717/peerj.2504)
Supplement: Supplemental Information 2 [file peerj-04-2504-s002.pdf]

Supplemental table S2. Description of primers used in the study

Primer	Sequence	Size range (bp)	Number of amplified alleles	Number of specific alleles
*Pul*01*	F: CAC CTT GTC CAC GGT TCT G<br>R: ACC AGG TCA GAG AGC TCA AC	161-176	15	3
*Pul*02*	F: TGA GTT CTT GCA CTT CAG GG<br>R: AAT CCC ACG AGT TAG TGC C	326-334	8	1
*Pul*04*	F: ACC GTT ACT GTC CAA CGG G<br>R: CCTGTA TGA ATG CAA CTT GAC G	219-234	15	1
*Pul*05*	F: GAT TAA TGG CGG GCG ACA G<br>R: TGG GTG CGC TAA TCG AGG	238-251	10	1
*Pul*06*	F: TGG CAT TCC TAG TTG ACG ATG G<br>R: GCT AGA CAA ACA AGA ATG CCT GC	161-170	8	0
*Pul*07*	F: ATC CCG AGG GAG AAT GCA C<br>R: AAG CAT GAG GTG TCT TGG C	328-338	10	2
*Pul*10*	F: TTT CCA AGC TCC GGC CAC<br>R: ACC GGT TGA GAC ACC CAA G	94-103	8	0
*Pul*11*	F: TCA ATC AAC CGC ATG TAG AGC<br>R: CAC GTG TAT TCG GCA GTC AG	296-337	18	0
Total			92	8

Primer	Sequence	Number of amplified bands	Number of specific bands
ISJ 4	5’ GTC GGC GGA CAG GTA AGT 3’	27	2
ISJ 5	5’ CAG GGT CCC ACC TGC A 3’	23	0
ISJ 11	5’ TGC AGG TCA AAC GTC G 3’	25	4
Total		75	6

*Szczecińska et al. (2013)
