# Supplementary material for: Comparison of the effectiveness of ISJ and SSR markers and detection of outlier loci in conservation genetics of Pulsatilla patens populations
Source: PeerJ. 2016 Nov 2;4:e2504. doi: 10.7717/peerj.2504 (PMC5101595; doi:10.7717/peerj.2504)
Supplement: Supplemental Information 3 [file peerj-04-2504-s003.pdf]

Supplemental table S3. Detection of outlier loci using *BayeScan*

	log10(PO)	qval	Alpha	F_{ST}
*Pul*04	1000.0	0.0000	-1.0899	0.14550
*Pul*06	2.8532	0.00046676	0.82574	0.48836
*Pul*10	1000.0	0.0000	-1.4054	0.11334
*Pul*11	1.9458	0.0031506	0.76520	0.47495
ISJ4_1	2.35454	0.00220044	1.1739	0.32552
ISJ4_6	2.0044	0.0047343	1.2266	0.33744
ISJ5_12	1.7672	0.0092418	1.0366	0.29756
ISJ5_13	1.8114	0.0073515	1.1604	0.32373
ISJ5_22	0.97782	0.023571	0.96673	0.28710
ISJ11_2	0.93332	0.035121	0.94879	0.28368
ISJ11_4	1000.0	0.0000	1.6179	0.42393
