# Supplementary material for: Comparison of the effectiveness of ISJ and SSR markers and detection of outlier loci in conservation genetics of Pulsatilla patens populations
Source: PeerJ. 2016 Nov 2;4:e2504. doi: 10.7717/peerj.2504 (PMC5101595; doi:10.7717/peerj.2504)
Supplement: Supplemental Information 4 [file peerj-04-2504-s004.pdf]

Supplemental table S4. Analysis of molecular variance (AMOVA) for *P. patens* populations

**Variation**		**df**	**SS**	**Variance**	**% total variance**	***p*-value**
	SSR				$\boldsymbol{F_{ST}}$	
Between populations		16	467.870	0.684	**20%**	0.001
Within a population		673	1890.842	2.810	**80%**	0.001
Total		689	2358.712	3.493	**100%**	0.001
					$\boldsymbol{\Phi_{PT}}$	
Between populations		16	935.740	2.503	**20%**	0.000
Within a population		328	3312.684	10.100	**80%**	0.000
Total		344	4248.423	12.603	**100%**	0.000
	ISJ				$\boldsymbol{\Phi_{PT}}$	
Between populations		16	682.044	1.855	**21%**	0.001
Within a population		325	2284.070	7.028	**79%**	0.001
Total		341	2966.114	8.883	**100%**	0.001

df – degrees of freedom, SS – sum of squares
