# Supplementary material for: Comparison of the effectiveness of ISJ and SSR markers and detection of outlier loci in conservation genetics of Pulsatilla patens populations
Source: PeerJ. 2016 Nov 2;4:e2504. doi: 10.7717/peerj.2504 (PMC5101595; doi:10.7717/peerj.2504)
Supplement: Supplemental Information 5 [file peerj-04-2504-s005.pdf]

Supplemental table S5. Genetic diversity F_{ST} (below diagonal) and R_{ST} (above diagonal) between the studied populations of *P. patens* based on all SSR loci

	**WI**	**PA**	**BB**	**PK**	**BL**	**PO**	**NS**	**KO**	**NM**	**NSz**	**NW**	**NP**	**GW**	**BO**	**B1**	**B2**	**B3**
**WI**		0.085	0.009	0.104*	0.483*	0.805*	0.313*	0.064*	0.439*	0.181*	0.450*	0.753*	0.861*	0.221*	0.368*	0.824*	0.750*
**PA**	0.203*		0.040*	0.081*	0.029	0.141*	0.056	0.127*	0.085*	0.138*	0.074	0.059	0.044	0.000	0.089	0.065*	0.069
**BB**	0.145*	0.165*		0.000	0.000	**0.024***	0.000	0.026*	0.007	**0.024***	0.009	0.007	0.003	0.065	0.313*	0.009	0.010
**PK**	0.287*	0.106*	0.215*		0.014	0.209*	0.058	0.000	0.122*	0.204*	0.121*	0.094*	0.094	0.133	0.291*	0.092*	0.096*
**BL**	0.366*	0.193*	0.307*	0.213*		0.742*	0.606*	0.019	0.602*	0.097*	0.683*	0.785*	**0.901***	0.079	0.220*	0.824*	0.693*
**PO**	0.262*	0.168*	0.185*	0.274*	0.393*		0.804*	0.085*	0.697*	0.455*	0.734*	0.708*	0.801*	0.409*	0.550*	0.663*	0.596*
**NS**	0.220*	0.258*	0.215*	0.335*	0.471*	0.307*		0.044	0.325*	0.222*	0.628*	0.820*	**0.901***	0.147	0.297*	0.876*	0.813*
**KO**	0.233*	0.115*	0.159*	0.221*	0.293*	0.081*	0.282*		0.066*	0.092*	0.070*	0.059*	0.060	0.188*	0.423*	0.058*	0.060
**NM**	0.233*	0.147*	0.136*	0.183*	0.283*	0.246*	0.305*	0.164*		0.086*	0.465*	0.777*	0.875*	0.243	0.398*	0.840*	0.765*
**NSz**	0.073*	0.128*	**0.069***	0.211*	0.280*	0.138*	0.134*	0.126*	0.162*		0.184*	0.488*	0.629*	0.387*	0.532*	0.509*	0.407*
**NW**	0.258*	0.176*	0.140*	0.240*	0.366*	0.242*	0.294*	0.200*	0.123*	0.159*		0.537*	0.734*	0.201	0.349*	0.732*	0.627*
**NP**	0.308*	0.172*	0.256*	0.361*	**0.475***	0.242*	0.436*	0.172*	0.335*	0.223*	0.341*		0.210*	0.152*	0.289*	0.405*	0.361*
**GW**	0.286*	0.139*	0.233*	0.345*	0.449*	0.201*	0.401*	0.144*	0.309*	0.190*	0.307*	0.341*		0.118*	0.253*	0.567*	0.549*
**BO**	0.185*	0.271*	0.254*	0.335*	0.445*	0.342*	0.294*	0.301*	0.339*	0.189*	0.375*	0.307*	0.380*		0.000	0.163*	0.175*
**B1**	0.278*	0.148*	0.224*	0.325*	0.406*	0.156*	0.405*	0.127*	0.291*	0.178*	0.289*	0.375*	0.150*	0.375*		0.296*	0.310*
**B2**	0.326*	0.165*	0.270*	0.351*	0.449*	0.190*	0.439*	0.154*	0.324*	0.221*	0.333*	0.289*	0.190*	0.443*	0.144*		0.018
**B3**	0.275*	0.127*	0.237*	0.322*	0.402*	0.194*	0.410*	0.156*	0.280*	0.194*	0.302*	0.333*	0.143*	0.385*	0.089*	0.191*	

* – statistically significant values ($p < 0.05$); values in bold – highest and lowest statistically significant values
