# Supplementary material for: Comparison of the effectiveness of ISJ and SSR markers and detection of outlier loci in conservation genetics of Pulsatilla patens populations
Source: PeerJ. 2016 Nov 2;4:e2504. doi: 10.7717/peerj.2504 (PMC5101595; doi:10.7717/peerj.2504)
Supplement: Supplemental Information 6 [file peerj-04-2504-s006.pdf]

Supplemental table S6. Genetic diversity Φ_{PT} between the studied populations of *P. patens* based on all ISJ loci

	**WI**	**PA**	**BB**	**PK**	**BL**	**PO**	**NS**	**KO**	**NM**	**NSz**	**NW**	**NP**	**GW**	**BO**	**B1**	**B2**	**B3**
**WI**																	
**PA**	0.309*																
**BB**	0.236*	0.170*															
**PK**	0.268*	0.187*	0.136*														
**BL**	0.262*	0.200*	0.184*	0.183*													
**PO**	0.198*	0.334*	0.184*	0.274*	0.213*												
**NS**	0.223*	**0.405***	0.208*	0.341*	0.315*	0.114*											
**KO**	0.309*	0.253*	0.157*	0.245*	0.217*	0.191*	0.273*										
**NM**	0.238*	0.186*	0.119*	0.202*	0.152*	0.155*	0.255*	**0.044***									
**NSz**	0.174*	0.228*	0.199*	0.230*	0.086*	0.157*	0.216*	0.153*	0.117*								
**NW**	0.108*	0.335*	0.166*	0.282*	0.242*	0.124*	0.116*	0.239*	0.198*	0.142*							
**NP**	0.254*	0.358*	0.179*	0.332*	0.325*	0.117*	0.237*	0.155*	0.152*	0.183*	0.148*						
**GW**	0.274*	0.291*	0.162*	0.297*	0.272*	0.245*	0.306*	0.198*	0.212*	0.219*	0.237*	0.236*					
**BO**	0.321*	0.227*	0.171*	0.207*	0.190*	0.270*	0.367*	0.160*	0.153*	0.175*	0.316*	0.306*	0.144*				
**B1**	0.259*	0.332*	0.183*	0.214*	0.259*	0.204*	0.281*	0.250*	0.254*	0.235*	0.199*	0.261*	0.121*	0.244*			
**B2**	0.329*	0.350*	0.240*	0.362*	0.352*	0.275*	0.336*	0.259*	0.283*	0.264*	0.303*	0.311*	0.000	0.217*	0.176*		
**B3**	0.237*	0.294*	0.231*	0.273*	0.268*	0.193*	0.315*	0.224*	0.221*	0.190*	0.238*	0.236*	0.101*	0.265*	0.134*	0.139*	

* – statistically significant values ($p < 0.05$); values in bold – highest and lowest statistically significant values
