# Supplementary figures and images for: Comparison of the effectiveness of ISJ and SSR markers and detection of outlier loci in conservation genetics of Pulsatilla patens populations
Source: PeerJ. 2016 Nov 2;4:e2504. doi: 10.7717/peerj.2504 (PMC5101595; doi:10.7717/peerj.2504)

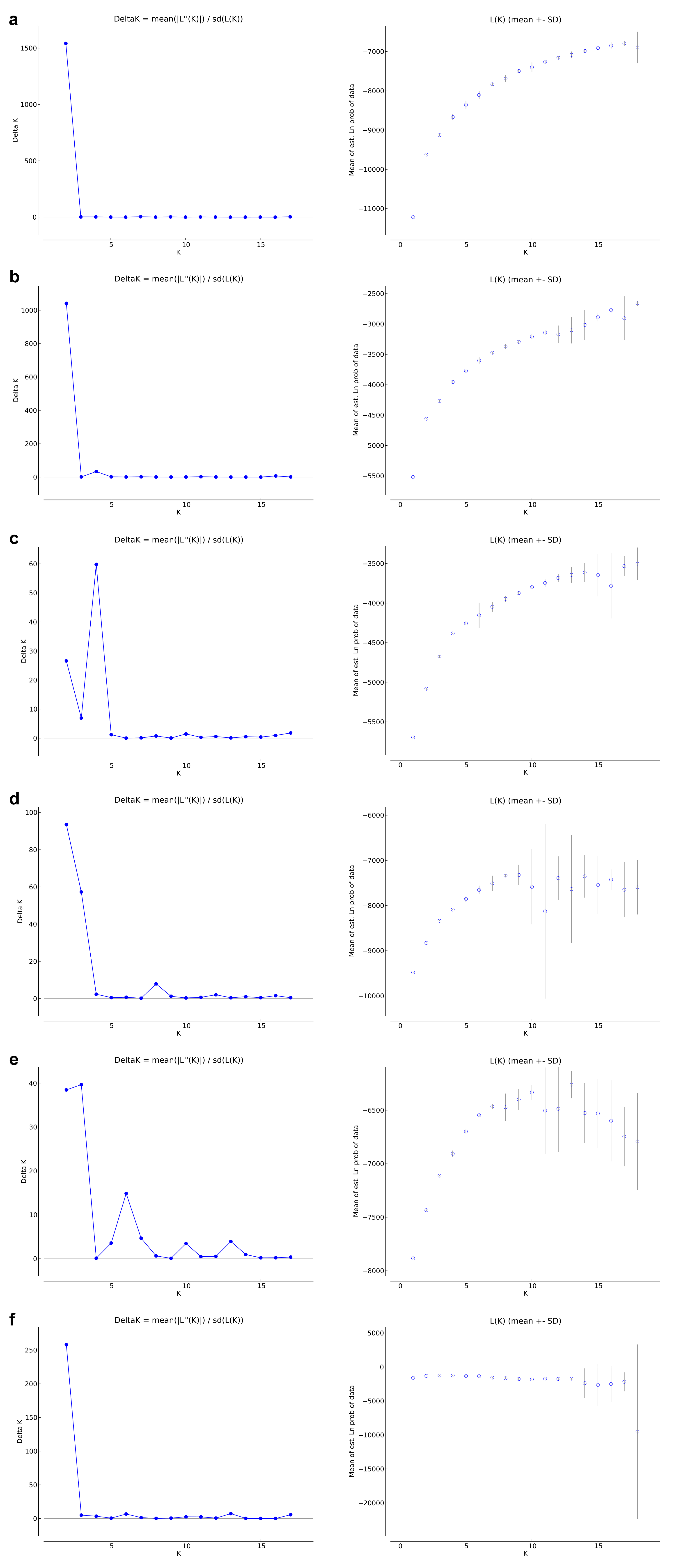

Supplement: Supplemental Information 7 — (A) based on all studied SSR loci; (B) based on neutral SSR loci; (C) based on outlier SSR loci; (D) based on all studied ISJ loci; (E) based on neutral ISJ loci; (F) based on outlier ISJ loci. [file peerj-04-2504-s007.png]
