# Supplementary material for: Comparison of the effectiveness of ISJ and SSR markers and detection of outlier loci in conservation genetics of Pulsatilla patens populations
Source: PeerJ. 2016 Nov 2;4:e2504. doi: 10.7717/peerj.2504 (PMC5101595; doi:10.7717/peerj.2504)
Supplement: Supplemental Information 8 — Populations with Q < 0.75 for each clusters are not included. [file peerj-04-2504-s008.pdf]

Supplemental table S8. Membership of particular populations to a specific cluster based on an arbitrary threshold of Q>0.75. Populations with Q<0.75 for each clusters are not included.

	Cluster 1	Cluster 2	Cluster 3	Cluster 4
All SSR loci (K=2)	WI, BB, PK, BL, NS, NM, NW, BO	PO, KO, NP, GW, B1, B2, B3	n/a	n/a
Neutal SSR loci (K=2)	WI, BB, PK, BL, NS, NM, NW, BO	PO, KO, NP, GW, B1, B2, B3	n/a	n/a
Outlier SSR loci (K=4)	WI, BO	NW	NP, GW, B1, B2, B3	PK, BL
All ISJ loci (K=2)	WI, PO, NS, NW,	PA, KO, NM, BO	n/a	n/a
Neutal ISJ loci (K=3)	NS, GW, B1, B2, B3		KO	n/a
Outlier ISJ loci (K=2)	WI, PO, NS, NW,	PA, KO, NM, GW, BO, B2	n/a	n/a

n/a- not applicable
